# Beyond reproduction: The ovary as a systemic regulator of female health and aging

**DOI:** 10.1371/journal.pbio.3003800

**Published:** 2026-05-27

**Authors:** Jennifer L. Garrison

**Affiliations:** Cellular and Molecular Pharmacology, University of California, San Francisco, California, United States of America; Harvard University T H Chan School of Public Health, UNITED STATES OF AMERICA

## Abstract

Classifying ovaries solely as reproductive organs has obscured their role as systemic regulators of female physiology. This Perspective discusses that ovarian aging is a primary determinant of healthspan and belongs at the center of geroscience.

## Teaser

Classifying ovaries solely as reproductive organs has obscured their role as systemic regulators of female physiology. This Perspective makes the case that ovarian aging is a primary determinant of healthspan and belongs at the center of geroscience.

Ovarian function directly impacts female health from childhood to old age. Ovaries undergo pronounced age-related functional decline earlier than most other tissues in the body, with systemic consequences that propagate across the brain, heart, liver, vasculature, immune system, bone, skin, and muscle over subsequent decades [1,2]. Female healthspan is compromised by ovarian aging, arguably among the most consequential and least studied processes in female biology. That this has not been a central focus of the aging field is surprising—less so once you notice that we spent the past century classifying ovaries as “reproductive” organs, a label that is accurate in the same way calling the pancreas a digestive organ is accurate: technically defensible, organized around one sole function, and deeply misleading about everything else it does.

Scientific vocabulary is rarely neutral. The words a field chooses encode assumptions about which questions matter and which disciplines own the problem. Classifying ovaries solely through the lens of reproduction has routed their biology through reproductive medicine and gynecology, disciplines organized around fertility and its clinical management. But ovaries are more than just egg factories. What ovaries do for the rest of the body, and what happens when that function degrades, are questions the field has not asked. Fertility occupies a roughly 30-year window of a woman’s life, but ovarian aging and its systemic effects shape health across the entire life span.

The brain offers an instructive parallel for how ovaries act at a systemic level. As the primary regulator of physiology and behavior, the brain functions as an integrating hub in a body-wide signaling network, coordinating the activity of tissues it never directly contacts via long-range signals that influence metabolism, immune function, and aging through mechanisms we are still mapping after decades of intensive work [3]. In female physiology, the ovaries maintain a bidirectional conversation with most tissues in the body through hormones and other circulating molecules, and that chemical conversation changes profoundly as ovaries age. We measure estrogen, progesterone, testosterone, and a handful of other ovarian hormones because they are the signals relevant to cycling and fertility. Whether they are the signals that matter most for female health and aging is a separate question. The ovary produces growth factors, cytokines, and other molecules that undergo poorly characterized age-related changes [4]; whether these, or others yet to be identified, drive aspects of systemic aging in females remains largely unexplored.

The dominant clinical and research paradigm has been woefully inadequate considering this impacts the health of half the world’s population. Ovarian function declines with age, a predictable cascade of pathologies follows, and the therapeutic response has been to replace estrogen (with or without progesterone) at mid-life in formulations that have changed little since the 1940s [5]. Menopausal hormone therapy is the best treatment available for the health risks that accompany menopause, and the renewed clinical enthusiasm for it is broadly justified [6]. But eight decades without many mechanistically novel solutions should prompt some reflection about whether the field has been asking the right questions. The consequences of ovarian aging are profound, and we need to discover what sets it in motion, why it happens so early, and why the timing of its decline varies so widely between individuals.

The field has also been looking at the wrong point in time—ovarian aging is not a perimenopausal phenomenon. Overt signs of ovarian aging, including declining follicle number, shifts in the hormonal, molecular, and physical environment of the ovary, and measurable changes in the signals it sends to other tissues, are detectable in women in their twenties and thirties [1]. Menopause is not the beginning of ovarian aging but closer to its end: the final clinically legible signal of a process that has been running for two to three decades by the time it becomes visible [7]. Organizing research around menopause as the primary exposure is a mistake. The underlying biology shows up decades earlier, and any window for understanding or intervening in it opens earlier still.

This reframing matters for how we interpret the data on aging we currently have. Large-scale analyses across tens of thousands of participants and thousands of circulating proteins have converged on a view of aging centered on inter-organ signaling coherence rather than cell-autonomous damage accumulation [8,9]. Organs age at different rates within individuals, and accelerated aging in one organ predicts disease in distant tissues. The biological ages of the immune system and brain are the strongest predictors of longevity because they are integrating nodes in a network, the coherence of which determines health trajectory. By every criterion that makes those organs interesting to researchers studying aging, the ovary belongs in this framework—yet it is absent from it entirely. The proteomic inflection these studies place at around age 60 in females is most plausibly a downstream consequence of ovarian changes that began accumulating decades earlier. We are measuring the echo, not the signal. Moreover, data on centenarians show that circulating proteins that distinguish individuals surviving to extreme old age cluster around immune regulation and metabolic homeostasis [8,9], the processes that ovarian signaling maintains across the life span and that deteriorate progressively as it declines. Whether female centenarians are disproportionately women whose ovarian aging was slow is a question the field has not asked and does not yet have the data to answer, partly because it has been studying the wrong end of the process and measuring the wrong things when it got there.

Language matters here: “reproductive aging” describes a fundamentally different process from “ovarian aging.” Reproductive aging refers to intrinsic aging in ovaries, fallopian tubes, and the uterus, leading to a decline in fertility and fecundity. Ovarian aging refers to the progressive functional deterioration of an endocrine and paracrine signaling organ with consequences that span the entire body. If ovaries are not functioning properly at any age, different health risks emerge. The clearest evidence that these are separable comes from women who lose ovarian function early: those who experience premature menopause, primary ovarian insufficiency, or surgical oophorectomy face substantially increased risk of cardiovascular disease, cognitive decline, osteoporosis, and metabolic dysfunction—not because they can no longer conceive, but because a signaling organ that has a pivotal role in systemic health has gone offline [10,11]. Conflating the two has routed a whole-body biology question through a subspecialty not designed to ask it, and influences where the research happens and who funds it.

The structural evidence for how thoroughly this question has been sidelined is not subtle; ovaries were absent from the original Human Cell Atlas and were added only years later [12,13]. The large longitudinal cohort studies underpinning the aging research field were built predominantly on males, with female biology as a secondary consideration at best and absent most of the time. Most multi-organ aging clocks do not include the ovary, despite its direct relevance to the inter-organ signaling story these datasets tell. Cohorts tracking ovarian aging phenotypes alongside systemic health outcomes across the female life span do not exist. How the disruption of ovarian signaling propagates across tissues over the decades before menopause is not known, because no study was designed to find out.

The large-scale omics platforms and analytical frameworks to address these questions exist, and preclinical models are evolving. Age at natural menopause is a key variable missing from large-scale clinical trial datasets and should be captured routinely to provide context for ovarian aging. What remains entirely absent is the epidemiological foundation of prospective cohorts that follow females from young adulthood, treating ovarian aging as a primary biological exposure rather than a footnote to reproductive history. Building that foundation is not a refinement of the existing geroscience research enterprise. It is the work the enterprise should have been doing all along.
